# How far does family physician supply correlate with district health system performance?

**DOI:** 10.4102/phcfm.v7i1.796

**Published:** 2015-06-19

**Authors:** Robin E. Dyers, Robert Mash, Tracey Naledi

**Affiliations:** 1 Division of Community Health, Faculty of Medicine and Health Sciences, Department of Interdisciplinary Health Sciences, Stellenbosch University, South Africa; 2 Health Programmes, Western Cape Government: Health, South Africa; 3 Division of Family Medicine and Primary Care, Faculty of Medicine and Health Sciences, Department of Interdisciplinary Health Sciences, Stellenbosch University, South Africa

## Abstract

**Background:**

Since 2011, a new cadre of family physicians, with 4 years of postgraduate training, was deployed in the district health services of the Western Cape, and tasked with a considerable range of duties aimed at a general improvement in care and health outcomes. There is a need to evaluate the contribution of these family physicians to the district health system.

**Aim:**

To develop a methodology for describing the correlation between family physician supply and district health system performance, clinical processes and outcomes, and to measure this correlation at baseline.

**Method:**

A cross-sectional study was undertaken that analysed data at an ecological level for the period of 01 April 2011 to 31 March 2012. This was a pilot project analysing data from the first year of a 4-year project. The correlations between family physician supply and 18 health system indicators were assessed within a logic model. The supplies of other categories of staff were also measured.

**Results:**

Although most of the correlations with family physicians were positive, the study was unable to demonstrate any strong or statistically significant correlations at baseline. There were significant correlations with other categories of staff.

**Conclusions:**

This study developed a methodology for monitoring the relationship between family physician supply using routinely collected indicators of health system performance, clinical processes and outcomes over time. Additional research will also be needed to investigate the impact of family physicians and triangulate findings as this methodology has many limitations and potential confounding factors.

## Abstrait

**A quel point le recrutement de médecins de famille va de pair avec la performance du système de santé des districts?**

**Contexte:**

Depuis 2011, un nouveau cadre de médecins de famille, avec 4 ans de formation après le Diplôme, ont été déployés dans les services sanitaires des districts du Western Cape, et chargés d’une série de fonctions dans le but d’améliorer les résultats de santé et de soins. Il faut évaluer la contribution de ces médecins de famille au système de santé des districts.

**Objectif:**

Développer une méthodologie pour décrire la corrélation entre le recrutement des médecins de famille et la performance du système sanitaire des districts, les processus et résultats cliniques, et mesurer cette corrélation à la base de référence.

**Méthode:**

Une étude transversale a été effectuée et a analysé les données au niveau écologique pour la période allant du 1er avril 2011 au 31 mars 2012. Ce projet pilote a analysé les données de la première année du projet de 4 ans. Les corrélations entre le recrutement des médecins de famille et les 18 indicateurs de systèmes de santé ont été évalués au sein d’un modèle logique. Le recrutement d’autres catégories de personnel a aussi été mesuré.

**Résultats:**

Bien que la plupart des corrélations avec les médecins de famille étaient positives, l’étude n’a pas pu démontrer de corrélations importantes ou statistiquement significatives à la base de référence. Il y avait des corrélations significatives avec d’autres catégories de personnel.

**Conclusion:**

Cette étude a mis au point une méthodologie pour évaluer la relation entre le recrutement des médecins de famille au moyen d’indicateurs de performance des systèmes sanitaires recueillis systématiquement, les processus et résultats cliniques avec le temps. Il faudra faire des recherches supplémentaires pour évaluer l’impact des médecins de famille et la triangulation des résultats étant donné que cette méthodologie a beaucoup de limitations et des facteurs de confusion potentiels.

## Background

Strengthening primary health care and district health systems has been recognised as one of the most important policy objectives for countries trying to improve health outcomes and equity.^1–3^ The 2008 World Health Report highlighted the ongoing need for this in its title ‘Primary health care: Now more than ever’ and also noted that most effective primary health care systems include doctors with a specialisation in either general practice or family medicine.^4^ Conversely they noted that many developing countries have developed primary health care in a limited and poorly resourced approach that is unlikely to succeed.

South Africa is seeking strategies to improve primary health care and district health systems with a view to introducing national health insurance in the longer term.^5^ The National Department of Health is considering what specialists are needed in the districts to improve the quality of care and health outcomes, especially in relation to maternal and child health.^5^

In South Africa, family medicine was recognised as a new area of specialisation in 2007 and training of family physicians as expert generalists began in 2008, with the first graduates in 2011. The national development plan states that ‘family physicians in the district specialist support team will take the primary responsibility for developing a district-specific strategy and an implementation plan for clinical governance’.^6^ Family medicine training is aligned with a set of national outcomes aimed at preparing these expert generalists to work independently at district hospitals and in primary care. The six roles of these new family physicians in the district health services (DHS) have been defined as providing clinical care, consulting on patients referred by other members of the health care team, mentoring and training other clinical staff, taking responsibility for clinical governance within the facility or subdistrict, supervising interns and registrars and also contributing to a more community-oriented approach.^7^ In general terms family physicians differ from medical officers in that they have 4 years of postgraduate training, have broader roles beyond clinical care and have greater length of experience.

Initial research on the impact of family physicians suggests that they have delivered on their roles as clinicians, consultants and leaders of clinical governance.^8^ Usually the family physician is the most senior member of the health care team in primary care or district hospitals and has an influence on the quality of care provided throughout the whole subdistrict. Interviews with district managers also suggest that they are impacting on key clinical processes for important conditions such as human immunodeficiency virus (HIV) infection, tuberculosis (TB), non-communicable diseases and childhood diarrhoea, as well as on the performance of the health services in terms of access to more comprehensive and coordinated care.^9^

Since 2011 the Western Cape has been the only province to employ family physicians at scale in district hospitals and community health centres and therefore provides an opportunity to evaluate the impact of family physicians on the DHS.^10^

Research in the USA and Iran has found a significant correlation between the supply of primary care physicians and better health outcomes, with an inverse relationship to the supply of other specialist doctors.^2,11,12,13,14,15,16,17^ This correlation, however, is potentially confounded by many other socio-economic factors such as standard of living, public education, access to information and economic empowerment of people.^15^

The aim of this study was to develop the methodology and to describe the baseline relationship between family physician supply and health system performance, key clinical processes and health outcomes in the DHS of the Western Cape, South Africa. No such work has previously been carried out in an African context. Given that the Western Cape was in the early stages of including family physicians in the DHS, this study focused on developing the methodology to correlate family physician supply with selected indicators from the health information system. Indicators were chosen in terms of the study's conceptual model, which saw the family physicians as a generic intervention that would eventually impact on health outcomes through an initial impact on health system performance and clinical processes. In this baseline study it was not anticipated that an actual impact would be measured.

This new knowledge is particularly relevant to the work currently being carried out by the National Department of Health on re-engineering primary health care, developing district specialist teams and planning for national health insurance.^5^ It will also be relevant to Ministries of Health and academic institutions in other African countries that are considering the training of family physicians (e.g. Botswana, Zimbabwe, Namibia, Kenya, Uganda and Rwanda).^18,19,20^

## Methods

### Study design

This is a cross-sectional ecological study that explores the baseline associations within a broader prospective study that ran from 2011 to 2014. This study focused on the first year from 01 April 2011 to 31 March 2012. The exploratory analysis on the baseline data of the larger study also served as a pilot of the methodology in the South African setting.

Data were collected from the existing health information system used by the Department of Health on the number of family physicians as of 2011, other health workers, key clinical processes, key health system functions, community indicators and health outcomes, and then aggregated to subdistrict and district level. The number of family physicians per 10 000 people was the measure of ‘supply’.

### Setting

The Western Cape Province is made up of six health districts: Cape Metropole, West Coast, Cape Winelands, Overberg, Eden and Central Karoo. The Cape Metropole, which represents the city of Cape Town, is further split into four substructures and each substructure is split into two subdistricts.

The Western Cape has ‘aligned its Comprehensive Service Plan with the model of having a family physician at each district hospital (> 50 beds) and each community health centre (> 30 000 people served)’.^9,21^ Although there were only about 20 family physicians (both old and newly trained) in the province in 2011, the overall perception was that they made a difference.^9^ Prior to 2011 family physicians were trained in part-time training programmes, which varied in terms of their learning outcomes and quality. Many of the family physicians employed by 2011 came from these earlier programmes.

As the new family physicians graduate from 2011 onwards, it is expected that the number of family physicians across the province will increase to between 60 and 80 over the next 5 years.^10^

The Western Cape was estimated to have a population of 5 755 607 in 2011 of whom approximately 83% were dependent on public health services. Relative to other provinces, the Western Cape had good access to basic amenities (e.g. 94% of households were electrified), but still had inequities within and between districts.^22,23^ The province shared the same quadruple burden of disease as the rest of the country: HIV-related disease and TB, interpersonal violence and trauma, maternal and child health problems and non-communicable chronic diseases.^24^

### Sample size

The units of analysis included the five rural districts and the eight Cape Town metropolitan subdistricts in the Western Cape. This mix of a total of 13 organisational units, described in Table 1, provided for similar-sized units for analysis. Whilst this is a small sample size, it is finite in that it includes collated data for the entire Western Cape geographic region for a period of 1 year.

**TABLE 1:** Description of units of analysis.

District or subdistrict	Total population	Dependent population	Percentage
Eden District	563 573	485 074	86
Central Karoo District	60 991	55 199	91
Cape Winelands District	768 295	652 148	85
Overberg District	238 086	207 202	87
West Coast District	314 926	270 949	86
Cape Metro Western Subdistrict	429 291	339 332	79
Cape Metro Southern Subdistrict	568 173	413 438	73
Cape Metro Northern Subdistrict	354 446	218 452	62
Cape Metro Eastern Subdistrict	445 037	360 936	81
Cape Metro Khayelitsha Subdistrict	427 157	410 104	96
Cape Metro Klipfontein Subdistrict	456 813	401 163	88
Cape Metro Mitchells Plain Subdistrict	521 966	462 453	89
Cape Metro Tygerberg Subdistrict	606 852	499 127	82

### Recruitment

The study included data on the Western Cape population (Table 1) and DHS. Data from central, specialised and regional hospitals were excluded. Private and community-based service data were also excluded.

### Inclusion criteria

Data from the following types of facilities within the DHS were included:

- clinics
- community day centres
- community health centres
- dental clinics
- district hospitals
- health posts
- midwife obstetrics units
- mobile services
- reproductive health services
- satellite clinics.

### Exclusion criteria

Data from the following types of facilities within the DHS were excluded:

- correctional services
- environmental health offices
- health promotion services
- home-based care
- non-medical sites.

Home-based care services, whilst part of DHS, were excluded because of differential procedures in data collection and resource allocation between the units of analysis in this study.

### Measurement tools

Data were collected in respect of the number of family physicians, other health workers, key clinical processes, key health system performance, community indicators and health outcomes.

**FIGURE 1:**
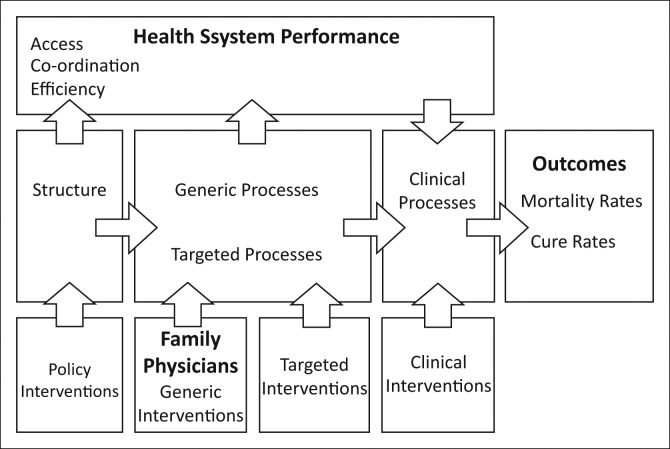
Modified Donabedian causal chain – Interventions at structural (policy) and generic service level can achieve effects through intervening variables further down the chain to result in particular health outcomes.^25^

A conceptual model that guided the evaluation and illustrates the inter-relationships between various elements is set out in Figure 1 and elaborated on below.^25^ The model, based on a modified Donabedian causal chain, was used to make sense of the complexity of the health system and to provide a rationale for the selection of indicators and identification of confounders. Indicators assessed were grouped according to the following categories:

**Policy intervention and structure:** Changes in the policy applied to the DHS were monitored qualitatively during the study.

**Generic interventions** such as human resources impact across a wide range of processes. In this case introducing a new cadre of family physicians was seen as a generic intervention. The study measured the number of family physicians and other practitioners (nurses, doctors and other specialists) in the DHS per 10 000 dependent population.

**Targeted interventions** were aimed at improving specific clinical processes via training, audit cycles or other clinical governance methods.

**Clinical interventions** directly impacted clinical processes through the provision of new drugs, devices, procedures or therapies.

**Health system performance:** Kringos et al. have identified key aspects of the primary care system upon which the family physician can be expected to have some impact.^26^ These include access to, continuity of, coordination of, comprehensiveness of, quality of and efficiency of care.

**Clinical processes:** Family physicians as expert generalists should impact across the full range of clinical processes.

**Health outcomes:** Key facility-based outcome indicators such as perinatal mortality.

### Definitions

Tarimo defines the DHS as a ‘well-defined population living within a clearly delineated administrative and geographic area. It includes all the relevant health care activities in the area, whether governmental or otherwise’.^27^ However, for this study the DHS definition was restricted to services rendered by the vertical funding programme 2: ‘District Health Services’ in the annual performance plan, which includes governmental primary health care facilities and district hospitals.10

Population-based indicators were expressed per ‘dependent population’ (Table 1). The dependent population is an estimate of the proportion of the population with insufficient household income to afford private medical care, whether by out-of-pocket payment or by medical insurance. It is therefore different from the ‘uninsured population’. Whilst the dependent population provides for a denominator of the population likely to utilise public health services, it can also be used as a proxy for deprivation.^28^

The final set of indicators that had to be collected was defined during the first 6 months of the project through a collaborative process between the researchers, current family physicians, district managers and the directorate for health impact assessment within the Western Cape Department of Health. Indicators were selected according to the conceptual framework (Figure 1) and in terms of their availability, credibility and expected impact by family physicians.

The aforementioned indicators were given shorter names as variables for convenience (Tables 2 and 3). For this study we chose four generic intervention variables (Table 2) and 18 proxy variables for clinical processes, health system performance and health outcomes (Table 3).

**TABLE 2:** Definitions of independent variables.

Independent variable	Definition
Family physician	Primary health care family physicians per 10 000 dependent population.
Nurse	Primary health care nurses per 10 000 dependent population; includes professional nurses, enrolled nurses and enrolled nursing assistants.
Medical officer	Primary health care medical officers per 10 000 dependent population.
Other specialist	Specialists employed by District Health Services, other than family physicians, per 10 000 dependent population; these include specialists in internal medicine, surgery, paediatrics, obstetrics and gynaecology, anaesthetics and orthopaedics.

**TABLE 3:** Definitions of dependent variables.

Dependent variable (framework domain)	Definition
PHC utilisation (access)	Rate at which primary health care services are utilised by the target population, represented as the average number of visits per person during the reporting period in the target population.^10^
Access (access)	Average score of questions related to primary health care access in the annual client satisfaction survey (scores ranged from −2 to 2).
Teamwork (coordination)	Average score of the question, ‘In my unit/component the staff function well as a team’ in the annual staff satisfaction survey (scores ranged from 1 to 5).
Chronic care team (coordination)	Proportion of facilities that have a designated chronic care team.
Hospital expenditure (efficiency)	Average cost (in South African rand) per patient day equivalent in district hospitals. Patient day equivalent is a weighted combination of inpatient days, day patients, and outpatient department and emergency headcounts; all hospital activity is expressed as an equivalent to one inpatient day.^10^
PHC expenditure (efficiency)	Expenditure on primary health care by the provincial Department of Health per dependent population; includes expenditure in primary health care facilities and district hospitals.
Cervical smears (clinical processes)	Proportion of women aged 30 years and older who have screening for cervical cancer.^10^
Isoniazid prophylaxis (clinical processes)	Proportion of HIV-positive patients started on isoniazid prophylaxis.
TB treatment (clinical processes)	Proportion of patients suspected of having TB who have started treatment.
CYPR (clinical processes)	Couple year protection rate: percentage women of reproductive age (15–44 years) who are using (or whose partner is using) a modern contraceptive method; contraceptive methods include female and male sterilisation, injectable and oral hormones, intrauterine devices, diaphragms, spermicides and condoms.^10^
Early antenatal booking (clinical processes)	Percentage of pregnant women who visit a health facility for the primary purpose of receiving antenatal care, often referred to as ‘a booking visit’, that occurs before 20 weeks after conception.^10^
Immunisation (clinical processes)	Percentage of all children under 1 year who complete their primary course of immunisation during the reporting period; a primary course includes BCG, OPV 0 & 1, DTaP-IPV-Hib 1, 2 & 3, HepB 1, 2 & 3, and first measles at 9 months.^10^
Diabetes score (clinical processes)	Aggregated Annual Chronic Disease Audit score for the questions about the clinical processes and the technical quality of care related to the management of patients with diabetes.
Hypertension score (clinical processes)	Aggregated Annual Chronic Disease Audit score for the questions about the clinical processes and the technical quality of care related to the management of patients with hypertension.
TB cure (outcomes)	Percentage of new smear-positive pulmonary tuberculosis cases cured at first attempt.^10^
Maternal mortality (outcomes)	Number of maternal deaths in facility expressed per 10 000 live births; a maternal death is the death of a woman whilst pregnant or within 42 days of termination of pregnancy, irrespective of the duration and the site of the pregnancy, from any cause related to or aggravated by the pregnancy or its management, but not from accidental or incidental causes (as cited in ICD 10).^10^
Perinatal mortality (outcomes)	Stillbirths plus the number of children who have died in a health facility between birth and 28 days of life, expressed per 10 000 total births in facility.
Under-5 mortality (outcomes)	The number of children who have died in a health facility between birth and their fifth birthday, expressed per 10 000 live births in facility.^10^

PHC, primary health care, TB, tuberculosis.

Staff numbers were expressed as ‘full-time equivalents’ (FTE) (Table 4). A FTE is a representation of the time spent by a particular staff category in rendering designated services during the total number of working hours for the financial year.

**TABLE 4:** Numbers of full-time equivalents per staff category in each district or subdistrict.

District or subdistrict	Family physician	Medical officer	Nurse	Other specialist
Eden District	1.00	54.26	720.17	9.66
Central Karoo District	0.00	12.68	174.89	0.00
Cape Winelands District	3.50	58.27	804.74	5.31
Overberg District	2.00	24.01	317.61	2.69
West Coast District	2.00	35.46	487.91	5.34
Western Cape Town Subdistrict	1.00	45.80	294.40	4.50
Southern Cape Town Subdistrict	1.00	89.05	515.93	22.21
Northern Cape Town Subdistrict	1.00	14.36	84.32	3.50
Eastern Cape Town Subdistrict	2.00	64.37	413.19	15.97
Khayelitsha Subdistrict	2.00	76.97	469.13	3.02
Klipfontein Subdistrict	2.00	89.56	506.88	23.67
Mitchells Plain Subdistrict	2.00	29.93	251.69	4.50
Tygerberg Subdistrict	1.00	91.69	625.43	13.90

### Data sources

Data were collected from routine health and human resource management information systems as follows:

**Persal:** human resource management tool, used to establish the numbers of ‘generic interventions’ such as various categories of staff.

**Sinjani:** routine monitoring and reporting tool, used to collate all health facility routine data in the province.

**ETR.net:** electronic TB registers, used to collate and report cohort data of TB patients.

**Chronic Diseases Audit:** annual provincially coordinated audit on the quality of care for chronic non-communicable diseases in primary health care facilities.

**Staff Satisfaction Survey:** biennial provincial audit of staff satisfaction.

### Statistical analysis

The Centre for Statistical Consultation at Stellenbosch University was consulted to assist with data analysis. Data for the 22 variables were collated using Microsoft Excel™ 2011. Data were then exported to and analysed in Stata™ version 13.1.

The mean and standard deviations (s.d.) were calculated for each of the variables. As this was an ecological study these would be the ‘means of means’. The s.d. was preferred to the 95% confidence interval to describe the variance in the data, rather than the precision of the means, as the results came from a finite data set.^29^ The median and interquartile ranges (IQR) were also calculated for each variable.

Simple correlation, Spearman's rho, was used to describe the relationship between the number of family physicians per 10 000 people and key health system performance, clinical processes and health outcomes for 2011. The socio-economic differences between subdistricts and districts were included by expressing population-based data according to ‘dependence’ (a measure of income inequality).

Data were analysed for all 13 organisational units. The level of significance chosen was p < 0.05. We undertook correlation analysis between the family physician supply and the 18 dependant variables listed in Table 3. Correlation values can be interpreted as:^30^

0.90–1.00 (−0.9 to −1.00) Very high positive (negative) correlation

0.70–0.90 (−0.70 to −0.90) High positive (negative) correlation

0.50–0.70 (−0.50 to −0.70) Moderate positive (negative) correlation

0.30–0.50 (−0.30 to −0.50) Low positive (negative) correlation

0.00–0.30 (0.00 to −0.30) Negligible correlation.

Graphs were generated using Stata™ version 13.1 to illustrate the relationship between the data points and the correlation (or regression) line. The data points were assigned unique colours and shapes to distinguish rural districts from urban subdistricts.

### Ethics considerations

This study was approved by the Health Research Ethics Committee (HREC) at Stellenbosch University, protocol number N11/10/012, and was conducted according to accepted and applicable national and international ethical guidelines and principles, including those of the International Declaration of Helsinki October 2008. Permission was obtained from the Provincial Health Research Committee (PHRC) to conduct the research and to provide the routine data required. Data used in this research were collated at the subdistrict level and did not involve individual patient identifiers.

## Results

Tables 5 and 6 present descriptive statistics for the independent and dependent variables, respectively, whilst Table 7 presents the results for the correlations between these variables.

**TABLE 5:** Descriptive statistics of independent variables (number of observations = 13 [organisational units]).

Independent variable	Mean (s.d.)	Median (IQR)	Range: maximum (minimum)
Family physician	0.04 (0.03)	0.05 (0.2–0.54)	0.1 (0)
Nurse	13.13 (6.75)	12.48 (11.44–14.85)	31.68 (3.88)
Medical officer	1.49 (0.58)	1.40 (1.12–1.88)	2.30 (0.65)
Other specialist	0.18 (0.15)	0.16 (0.10–0.28)	0.52 (0)

s.d., standard deviation; IQR, interquartile ranges.

**TABLE 6:** Descriptive statistics of dependent variables (number of observations = 13 [organisational units]).

Dependent variable (framework domain)	Mean (s.d.)	Median (IQR)	Range: maximum (minimum)
PHC utilisation (access)	3.29 (0.49)	3.37 (2.95–3.70)	4.05 (2.54)
Access (access)	0.20 (0.32)	0.20 (0.03–0.44)	0.70 (–0.43)
Teamwork (coordination)	0.73 (0.18)	0.77 (0.63–0.88)	1.00 (0.41)
Chronic care team (coordination)	0.51 (0.27)	0.54 (0.36–0.67)	1.00 (0)
Hospital expenditure (efficiency)	1475.37 (453.06)	1772.59 (1273.67–1772.59)	2272.16 (728.97)
PHC expenditure (efficiency)	813.17 (360.03)	804.66 (620.24–901.42)	1644.59 (280.62)
Cervical smears (clinical processes)	0.76 (0.17)	0.77 (0.70–0.78)	1.11 (0.46)
Isoniazid prophylaxis (clinical processes)	0.09 (0.10)	0.07 (0.19–0.15)	0.35 (0.01)
TB treatment (clinical processes)	0.88 (0.06)	0.89 (0.84–0.90)	0.95 (0.76)
CYPR (clinical processes)	0.45 (0.09)	0.43 (0.41–0.47)	0.62 (0.32)
Early antenatal booking (clinical processes)	0.57 (0.11)	0.54 (0.49–0.68)	0.71 (0.41)
Immunisation (clinical processes)	0.88 (0.13)	0.87 (0.80–0.91)	1.18 (0.71)
Diabetes score (clinical processes)	0.48 (0.13)	0.49 (0.45–0.54)	0.68 (0.22)
Hypertension score (clinical processes)	0.53 (0.16)	0.55 (0.51–0.59)	0.78 (0.23)
TB cure (outcomes)	0.81 (0.05)	0.85 (0.79–0.86)	0.90 (0.73)
Maternal mortality (outcomes)	3.32 (4.59)	1.73 (0–3.88)	11.38 (0)
Perinatal mortality (outcomes)	140.05 (70.91)	112.61 (91.72–185.66)	272.23 (40.03)
Under-5 mortality (outcomes)	48.83 (52.49)	46.10 (3.65–92.71)	166.05 (0)

s.d., standard deviation; IQR, interquartile ranges; PHC, primary health care, TB, tuberculosis.

**TABLE 7:** Spearman's rho correlations between independent variables (Table 2) and dependent variables (Table 3).

Dependent variable (framework domain)	Family physician	Medical officer	Nurse	Other specialist
	Correlation coefficient(p-value)	Correlation coefficient (p-value)	Correlation coefficient (p-value)	Correlation coefficient (p-value)
PHC utilisation (access)	−0.27 (0.37)	0.18 (0.55)	0.09 (0.76)	−0.27 (0.36)
Access (access)	−0.13 (0.70)	−0.24 (0.46)	−0.19 (0.56)	−0.79* (< 0.01)
Teamwork (coordination)	0.09 (0.76)	0.27 (0.37)	0.47 (0.10)	0.19 (0.54)
Chronic care team (coordination)	0.38 (0.20)	−0.33 (0.27)	−0.83* (< 0.01)	−0.02 (0.94)
Hospital expenditure (efficiency)	−0.13 (0.70)	0.78* (< 0.01)	0.24 (0.44)	0.25 (0.43)
PHC expenditure (efficiency)	0.04 (0.90)	0.45 (0.13)	0.98* (< 0.01)	0.10 (0.73)
Cervical smears (clinical processes)	0.09 (0.76)	−0.33 (0.26)	0.25 (0.42)	-0.54 (0.05)
Isoniazid prophylaxis (clinical processes)	0.13 (0.68)	−0.13 (0.67)	0.56* (0.04)	−0.41 (0.16)
TB treatment (clinical processes)	0.09 (0.77)	−0.17 (0.58)	0.34 (0.25)	−0.10 (0.74)
CYPR (clinical processes)	0.32 (0.28)	0.00 (1.00)	0.45 (0.14)	−0.54 (0.05)
Early antenatal booking (clinical processes)	0.23 (0.45)	−0.47 (0.11)	0.50 (0.08)	−0.20 (0.52)
Immunisation (clinical processes)	0.03 (0.93)	−0.19 (0.53)	−0.08 (0.79)	0.37 (0.20)
Diabetes score (clinical processes)	−0.09 (0.76)	−0.25 (0.41)	−0.95* (< 0.01)	0.01 (0.96)
Hypertension score (clinical processes)	−0.15 (0.65)	−0.24 (0.42)	−0.84* (< 0.01)	0.23 (0.45)
TB cure (outcomes)	0.19 (0.53)	0.09 (0.94)	−0.33 (0.27)	0.24 (0.42)
Maternal mortality (outcomes)	−0.07 (0.83)	−0.08 (0.76)	−0.25 (0.41)	0.28 (0.34)
Perinatal mortality (outcomes)	0.17 (0.58)	−0.06 (0.85)	0.66* (0.01)	−0.26 (0.38)
Under-5 mortality (outcomes)	−0.01 (0.97)	0.06 (0.84)	0.67* (0.01)	−0.37 (0.21)

PHC, primary health care, TB, tuberculosis.

*, Statistically significant correlation (*p* < 0.05)

Statistically significant correlations (see Table 7) could not be demonstrated at baseline in 2011 for family physician supply and the 18 dependent variables within the aforementioned framework (Figure 1).

A significant positive correlation was observed between medical officers and hospital expenditure as well as between nurses and primary health care (PHC) expenditure (Table 7). Nurses were significantly correlated with the presence of a chronic care team, isoniazid prophylaxis for TB, higher perinatal and under-5 mortality, as well as lower audit scores for diabetes and hypertension care. The presence of other specialists was significantly correlated with lower access to care.

**FIGURE 2:**
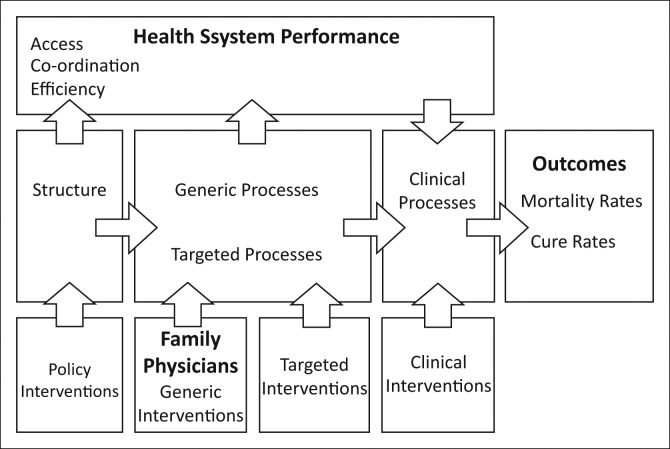
Scatter plot and regression line illustrating the correlation between family physician supply and hypertension scores in the annual chronic disease audit of District Health Services facilities.

**FIGURE 3:**
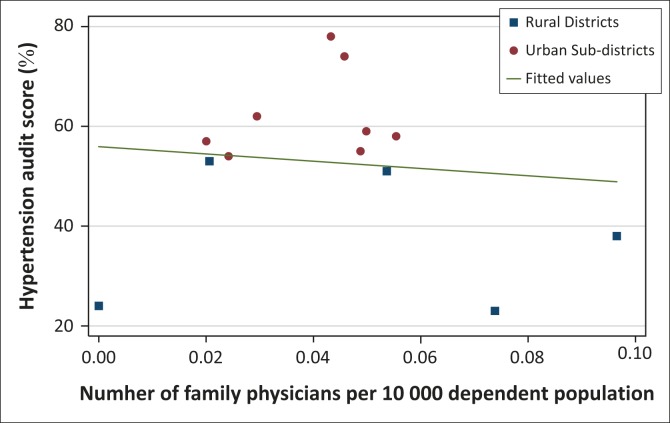
Scatter plot and regression line illustrating the correlation between family physician supply and the percentage of HIV-positive patients started on isoniazid prophylaxis for tuberculosis.

Figures 2 and 3 illustrate the scatter of data between urban and rural data points and regression lines.

## Discussion

The study did not find a strong baseline relationship between the supply of family physicians and the selected indicators of health system performance, clinical processes and outcomes. Whilst most of the correlations between family physicians and selected indicators within the conceptual framework (Figure 1) were positive, they were weak in magnitude. As this study reports data at baseline, during the first year that specialist family physicians were available to the DHS, it is not surprising that no strong relationship could be found. In addition, although the study made use of data from the whole province, the small number of units of analysis will have reduced the power of the study to show a statistically significant relationship.

The segregation of data points between rural and urban subdistrict or district in the graphs (Figures 2 and 3) is an indication that there may be confounding factors linked to the degree of rurality. These factors were mentioned in the adjustment models of previous large studies from the USA.2,11,12 Vogel and Ackerman pointed out that there was significant variation in the population in terms of composition and determinants of health beyond those that were measured and adjusted for in previous research.^14,31^

The correlations in Table 7 may be confounded by known and unknown factors. Access and health outcome variables are particularly prone to confounding by social determinants of health beyond the immediate scope of the family physicians. Given that family physicians’ roles include improving clinical governance and teaching junior staff, one would expect districts with higher supplies of family physicians to perform better in audits of clinical processes and in coordination of care.

### Limitations of the study design

The issue of temporality is an important limitation of this cross-sectional ecological study. These correlations cannot determine the direction of any underlying cause and effect between the variables. Therefore causality between independent and dependent variables cannot be assumed.

As tempting as it may be, we cannot say that the significant negative association between other specialists and access is in keeping with Shi and Starfield's findings that specialist-dominated health systems have poorer performance.^12,13^

This was an observational study design with no control group. It was merely a ‘dose-response’ type analysis on an ecological level. This means that any findings, whether positive, negative or inconclusive, cannot be inferred to the level of the individual.

The significance of correlation co-efficient is of limited value with a small sample size.^32^ The absence of statistical significance does not necessarily exclude the existence of correlations that could be regarded as important in this policy context.

Given that this study used a finite sample, some may argue that the magnitude of correlations observed can be taken at face value, regardless of the statistical significance. However, those correlations were observed for a period of only 1 year within the province, which limits the validity of non-significant findings beyond that period and beyond the Western Cape borders.

The Spearman's rank correlation test examines correlation between the indicator and the rank of the number of family physicians per population rather than the actual number of family physicians per population. Therefore, it does not factor in dramatic differences in the magnitude of the independent variable.

The selection of indicators was affected by deficiencies in the availability or reliability of data and therefore some indicators which might have been important in terms of the family physicians’ impact could not be used.

## Recommendations

This study has developed a methodology that can be repeated for subsequent years (2012–2014) to monitor how the relationships evolve over time and with an increasing number of family physicians.

Further research using an experimental or quasi-experimental design is needed to investigate the impact of family physicians on health outcomes. Findings from surveys and qualitative research methods^9^ may also be used to determine whether family physicians are performing the envisioned tasks, whilst longitudinal studies may demonstrate their impact on generic, targeted and clinical processes. Cost-effectiveness of family physicians may also be evaluated in future studies.

## Conclusion

We were unable to demonstrate strong correlations between family physician supply and clinical processes, health system performance or facility-based outcomes at the baseline of this ongoing study, but we were able to develop the methodology and to illustrate the presence of confounding factors that were not included in similar research in other settings. Whilst it was arguably too early to show a strong correlation, this study also highlighted the limitations of this study design. Care should be taken not to assume causality within relationships found from such a design. In future it may be necessary to perform additional complementary research in order to triangulate and understand any emerging relationships.
